# The Effects of Feeding *ybfQ*-Deficient Gut Bacteria on Radio-Tolerance in Symbiotic *Caenorhabditis elegans*: The Key Role of Isoscoparin

**DOI:** 10.3390/microorganisms13112626

**Published:** 2025-11-19

**Authors:** Liu Ding, Jingjing Zhang, Shanpeng Qiao, Jiyu Xu, Jing Li, Wenjing Zhang, Qiyi Yi, Yuejin Wu, Ting Wang, Po Bian

**Affiliations:** 1 Hefei Institutes of Physical Science, Chinese Academy of Sciences, Hefei 230031, China; 2 Science Island Branch of Graduate School, University of Science and Technology of China, Hefei 230026, China; 3 Teaching and Research Section of Nuclear Medicine, School of Basic Medical Sciences, Anhui Medical University, 81 Meishan Road, Hefei 230032, China

**Keywords:** gut microbiota, oxidative stress, radio-tolerance, isoscoparin, *Caenorhabditis elegans*

## Abstract

It is inevitable for life on earth to be exposed to various types of ionizing and non-ionizing radiation, underscoring the importance of radioprotection. The symbiotic interaction between gut microbiota and the host provides a strategy for protecting the organism against these stressors. However, the genetic mechanisms underlying this interaction remain poorly understood due to the complexity and diversity of gut microbiota. In this study, we employed a symbiotic experimental system involving *Caenorhabditis elegans* and *Escherichia coli* to systemically investigate the effects of bacterial genetic alterations on host responses to radiation exposure. Our findings revealed that deletion of the bacterial *ybfQ* gene (*ΔybfQ*) significantly enhanced worm tolerance to UV-B radiation. Transcriptomic analysis demonstrated an enhanced antioxidant capacity in *ΔybfQ*-fed worms, as evidenced by reduced levels of reactive oxygen species (ROS) and restored oxidative homeostasis. Notably, *ΔybfQ* bacteria exhibited overproduction of isoscoparin, and exogenous supplementation with isoscoparin similarly enhanced worm radio-tolerance, underscoring its crucial role in *ΔybfQ*-mediated antioxidant of host worm. Both interventions retained their protective effects in IIS-deficient worms (*daf-16*). However, the protective effects of *ΔybfQ* feeding, but not isoscoparin treatment, were attenuated in *daf-2* worms with a constitutively activated IIS pathway, accompanied by reduced bacteria gut colonization. Collectively, our results provide novel insights into the genetic basis of host-microbe interactions and propose a potential pharmacological strategy for radiation protection.

## 1. Introduction

Intensified damage to ozone layer from modern human activities has led to increased levels of UV radiation reaching the earth surface. This phenomenon poses a significant threat to human health and elevates it to a critical global public health concern [1,2]. UV radiation not only causes skin damage and increases the risk of skin cancer, but also suppresses immune function, thereby diminishing the body’s ability to combat infections and increasing susceptibility to certain diseases [3,4]. Nevertheless, moderate exposure to sunlight remains essential for various physiological processes, particularly as the primary source cutaneous vitamin D synthesis [5]. Therefore, there is a pressing need to develop effective protective agents to mitigate these potential health risks. Recent studies have identified several compounds, such as salvianolic acid B, dietary foeniculum vulgare mill extract, and *Juglans regia* L, which demonstrate the ability to alleviate UV-induced skin damage [6,7,8]. However, further research is required to develop more potent UV-protective agents. Emerging evidence has underscored the protective role of the gut microbiota in managing environmental stress [9], suggesting a promising avenue for identifying bacteria or their metabolites that may safeguard the host against UV-related health risks.

The gut microbiota plays a critical role in maintaining human health and represents the largest bacterial community within the human body [10,11,12]. A symbiotic interaction exists between gut microbiota and their hosts, wherein the host supplies nutrition and a stable environment for bacterial growth, while the microbiota contributes by facilitating energy extraction from indigestible dietary components and synthesizing essential metabolites [13,14,15]. These metabolites originate from three primary sources: exogenous intake, host-derived substances modified by gut bacteria, and de novo synthesis by microbiota itself [16,17]. They can enter the systemic circulation through multiple pathways, with studies estimating that approximately 10% of circulating metabolites in mammalian blood are derived from gut microbial activity [18,19,20,21]. These microbial metabolites perform vital functions in regulating host immunity, metabolism, nervous system function, and overall physiological homeostasis [22,23,24,25]. Imbalance in this symbiotic relationship has been associated with various non-communicable disorders such as diabetes, inflammatory bowel disease, and obesity [26,27,28].

Emerging evidence underscores the protective role of the gut microbiota against radiation-induced damage. The gut is a rapidly regenerating tissue and serves as a major target organ for radiation injury. Accumulating evidence indicates that gut microbiota and their metabolites play a critical role in mitigating radiation-induced injury to the gut [29,30,31,32]. Notably, this protective effect extends beyond the gut to benefit the entire host organism. For example, gut microbiota isolated from mice that survived high-dose X-ray exposure have been shown to enhance radio-resistance when transplanted into the gut of naïve mice [9]. Under normal physiological conditions, the gut bacteria maintain a relative equilibrium [33,34]. However, it is highly susceptible to various factors, particularly radiation exposure. Studies have demonstrated that radiation exposure significantly alters the composition of the gut microbiome, leading to a reduction in beneficial bacteria and an increase in pathogens and opportunistic pathogens [35,36]. Moreover, the radiation exposure affects the gut microbiota at the genetic level; however, there is limited evidence regarding the specific role of these genetic alternations in modulating host’s response to radiation.

The complexity and diversity of the gut bacteria in mammals and humans pose substantial challenges in elucidating the role of genetic alternations. *Caenorhabditis elegans* (*C. elegans*) is widely acknowledged as a model organism across various domains of life sciences, including radiation biology. Its simple dietary requirements make it an ideal system for investigating the symbiotic interaction between a single type of gut bacteria and the host organism [37,38]. Additionally, the Keio collection, consisting of 3985 *E. coli* strains with single-gene knockouts of non-essential genes, is readily available [39,40]. Consequently, the symbiotic relationship between *C. elegans* and *E. coli*, in conjunction with the Keio collection, offers a high-throughput genetic platform for screening and identifying bacterial mutants that elicit specific physiological effects on the host worms.

In this study, we developed a symbiotic experimental model involving worms and *E. coli* for a UV-protective screening assay. The worms were fed various *E. coli* strains from the Keio collection and subsequently exposed to UV-B radiation, allowing for the identification of bacterial strains that confer significant radio-protective effects. A total of 65 positive strains were identified through this screening process. Among these, a strain harboring a deletion of the *ybfQ* gene (*ΔybfQ*) was selected for further investigation. The results indicated that the overproduction of isoscoparin in the *ΔybfQ* bacteria significantly contributes to the observed radiotolerance in the worms. Furthermore, the exogenous administration of a plant-derived analog produced a similar beneficial effect.

## 2. Materials and Methods

### 2.1. C. elegans Culture and Synchronization

The *C. elegans* strain N2 (Bristol) was utilized for general experiments. The following mutant strains were employed: DA1116: *eat-2(ad1116)II*, CF1553: *muIs84[(pAD76) sod-3p::GFP+rol-6(su1006)]*, GA186: *sod-3(tm760)X*, CF1038: *daf-16 (mu86)I*, and CB1370: *daf-2 (e1370)III.* All strains were obtained from Caenorhabditis Genetics Center (CGC). Culturing and handling of the strains adhered to the standardized protocols outlined by Brenner [41]. Briefly, worms were maintained at 20 °C on nematode growth media (NGM) plates supplemented with BW25113 (BW) or other strains. Synchronized larvae were generated according to previously described methods [42].

### 2.2. Worm Radiation Exposure

**Figure 1:**
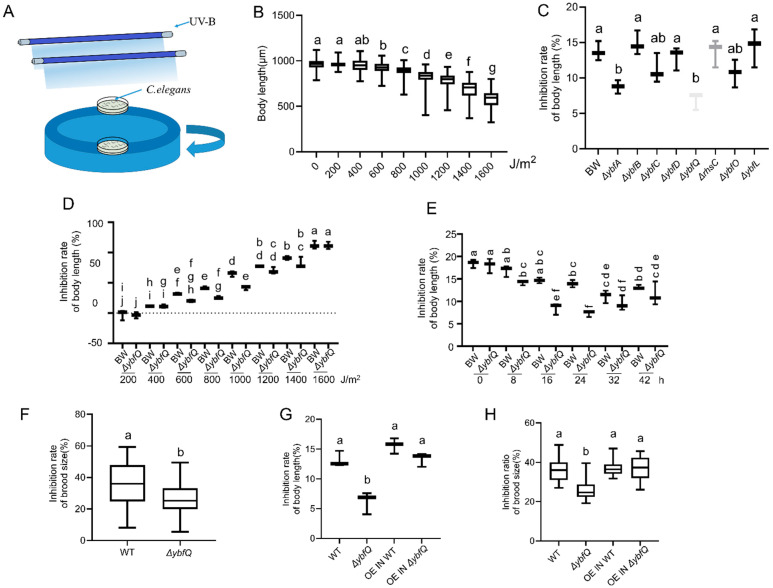
Impact of the *ybf* genes mutation in *Escherichia coli* on radio-tolerance in *Caenorhabditis elegans*. (**A**) Schematic diagram of the UV-B irradiation setup; (**B**) Dose–response relationship between UV-B irradiation and worm growth inhibition (n = 5); (**C**) Effect of the *ybf* genes mutation on UV-B-induced growth inhibition (n = 3); (**D**) Dose–response relationship between BW25113 (BW) and *ΔybfQ* on worm growth inhibition (n = 3); (**E**) UV-B-induced growth inhibition at the indicated worm ages (n = 3); (**F**) Brood size of worms exposed to 800 J/m^2^ at age of 24 h (n = 5); (**G**) Effect of the expression of the wild-type *ybfQ* gene in *ΔybfQ* and BW25113 (BW) strains on UV-B-induced growth inhibition (n = 3); (**H**) Effect of the expression of the wild-type *ybfQ* gene in *ΔybfQ* and BW strains on worm brood size (n = 5). Significant differences (*p* < 0.05) among experimental groups are indicated with different letters.

Worms of specified ages were transferred to 35-mm NGM plates devoid of bacterial lawns. UV-B exposure was administered using two 312 nm light sources (TL 20W/01-RS, Philips, Amsterdam, The Netherlands) with an intensity of 200 μW/cm^2^. The worms were placed on a rotating disk to ensure homogenous radiation (Figure 1A). The UV-B intensity was measured using an ultraviolet radiation meter (LS125, Linshang, Shenzhen, China). X-ray radiation was conducted using a SHARP 100 irradiator (Raycision, Hefei, China) at a dose rate of 12.5 Gy/min (225 KeV). Carbon ion irradiation was conducted at the Heavy Ion Research Facility in Lanzhou, China, with an energy of 80 MeV/u and dose rate of 80 Gy/min. For all irradiation experiments, worms were prepared under standard laboratory conditions, transported to the respective irradiation facility (a process completed within one hour), and subsequently returned for further culture and analysis.

### 2.3. Worm Chemotaxis and Dietary Assays

To assess preference of worms for BW25113 versus *ΔybfQ* bacteria, we conducted chemotaxis experiments as previously described [43]. Briefly, two groups of bacteria were positioned diagonally on opposite sides of a 90-mm NGM plate. The worms were then transferred to the center of the plate. After 2 h, the number of worms that reached each bacterial lawn were counted, as detailed in Supplementary File Figure S1A. Worm dietary intake was evaluated using the pharyngeal pumping assay. The number of pharyngeal pumps per minute was recorded, with 30 worms examined per experiment.

### 2.4. Analysis of Worm Radio-Tolerance

To evaluate worm radio-tolerance, growth metrics were measured. Prior to radiation, the baseline body lengths of the worms were recorded. Body lengths were recorded again at 24 h post-radiation, with a minimum of 30 worms examined per experiment. Imaging was conducted using a stereomicroscope (30×, SMZ171, Motic, Xiamen, China), and body lengths were quantified using ImageJ 1.x software (https://imagej.net/ij/, accessed on 23 November 2018). The inhibition rate was calculated as follows: (final length of control group − final length of the treated group)/baseline body length. Additionally, brood size served as another endpoint for assessing radio-tolerance. Following radiation, ten worms from each group were individually transferred to separate plates and allowed to lay eggs over a period of five days. The total number of larvae and eggs was subsequently counted.

### 2.5. Assay of Oxidative Stress in Worms

ROS levels in worms were assessed using the fluorescent dye carboxy-H2DCFDA (S0035S, Beyotime, Shanghai, China). Specifically, worms were co-incubated with 100 µM H2DCFDA in the dark at 20 °C for 2 h. Following incubation, the worms were washed and imaged using fluorescence microscopy (AX10, ZEISS, Jena, Baden-Wurttemberg, Germany). Additionally, to further evaluate oxidative status, the transgenic worm strain CF1533 (SOD-3::GFP) was utilized. The *sod-3* gene encodes an iron/manganese superoxide dismutase, which is a key antioxidant enzyme in worms. After radiation, relative GFP signals were quantified using ImageJ software. Each experiment included an examination of 30 worms.

### 2.6. Worm Transcriptomic Analysis

mRNA isolation was performed using the NEBNext^®^ Poly(A) mRNA Magnetic Isolation Module Kit (E7490S, New England Biolabs, Ipswich, MA, USA). RNA-seq libraries were prepared with the KAPA Stranded RNA-Seq Library Prep Kit (KK8420, Roche, Basel, Switzerland). Transcriptomic profiling and data analysis were conducted at Aksomics (Shanghai, China). Genes exhibiting a fold change greater than 2.8 and a *p*-value less than 0.05 were identified as significant differences.

### 2.7. Bacterial Growth and Manipulation

A single bacterial colony was inoculated into LB medium and grown at 37 °C for 6 h under shaking conditions at 200 rpm. Subsequently, 150 μL of the bacterial culture was uniformly spread onto a 60-mm NGM plate and allowed to dry overnight before worm placement. The bacterial heat-killing experiments were performed following an established protocol [44]. For supernatant exchange experiments, bacterial cultures were centrifuged to separate the supernatant from the bacterial pellet. The supernatant was subsequently filtered 2–3 times through 0.22 µm filters to ensure complete removal of any residual bacterial cells. The bacterial pellet was then re-suspended in the supernatant obtained from a different bacterial culture. Isoscoparin (HY-N5080, MedChemExpress, Monmouth Junction, NJ, USA), a natural flavonoid isolated from Gentiana algida Pall, was dissolved in DMSO to prepare a stock solution at a concentration of 10 mg/mL, which was subsequently diluted with LB medium to obtain the working solution. Bacterial phenotype analysis was conducted as follows: Bacteria in the logarithmic growth phase were diluted in LB medium. After staining with ammonium oxalate crystal violet, images of the bacteria were captured using an AX10 microscope (ZEISS), and the cell sizes were quantified using ImageJ software. Bacterial gut colonization was assessed using a previously described method [45].

### 2.8. Establishment of Transgenic Bacterial Strains

The *ybfQ* gene was amplified from BW bacteria via PCR. Following verification and purification using the SanPrep Column PCR Product Purification Kit (Cat. No. B518141, Sangon Biotech, Shanghai, China), the amplicons were ligated into the pEASY^®^-Blunt E1 Expression Vector (Cat. No. CE111-01, TransGen Biotech, Beijing, China) and subsequently transformed into Trans1-T1 Phage Resistant Chemically Competent Cells (Cat. No. CD501, TransGen Biotech). The transformed cells were then plated on LB plate supplemented with ampicillin (50 mg/mL) to select for positive colonies. Plasmids were extracted from these colonies using the SanPrep Column Plasmid Mini-Preps Kit (Cat. No. B518191, Sangon Biotech) and introduced into competent cells of both BW and *ΔybfQ* bacterial strains to generate overexpression lines. To visualize bacterial gut colonization, we generated a GFP-reporter bacterial strain by transforming them with the pUC19-EGFP plasmid, which carries a *lac::gfp* fusion, using the protocol described above.

### 2.9. Bacterial Metabolomics and Transcriptomic and Analysis

Metabolomics analysis was conducted at BioMarker Technologies (Beijing, China) to characterize the metabolic profile of the bacterial samples. The sample preparation protocol was as follows: Metabolites were extracted with the extraction solution of the internal standard (with a volume ratio of methanol to acetonitrile of 1:1 and an internal standard concentration of 20 mg/L), and treated with a 45 Hz grinding instrument for 10 min and then ultrasonicated for 10 min. The resulting extracts were then centrifuged at 14,000× *g* rpm for 15 min at 4 °C to remove protein and cellular debris. The supernatant was carefully collected, vacuum-dried, and reconstituted in Extract solution (volume ratio of acetonitrile to water: 1:1) for instrumental analysis. Extraction solvent was added into the samples, followed by high-performance liquid chromatography (UltiMate 3000, ThermoFisher, Waltham, MA, USA) coupled with high-resolution mass spectrometry (Orbitrap Exploris 480, ThermoFisher). Data processing and analysis were performed using the CD search Library software (Compound Discoverer 3.3.3.200). Transcriptomic analysis was conducted at BioMarker (Beijing, China). The procedures included the extraction of total RNA, removal of rRNA, fragmentation of mRNA, reverse transcription to synthesize cDNA, adaptor ligation, UNG enzyme-mediated digestion of the second strand of cDNA, and subsequent sequencing. The library was constructed using the TruSeq Stranded Total RNA Library Prep Kit (Cat. No. RS-122-2201, Illumina, San Diego, CA, USA).

### 2.10. Quantification and Statistical Analysis

All experiments were independently conducted in triplicate or more. Data analysis was performed using GraphPad Prism 9 software (GraphPad Software Inc., San Diego, CA, USA). The Shapiro–Wilk test confirmed that all quantitative data sets satisfied the assumption of normal distribution. Therefore, ANOVA was employed, followed by Tukey’s post hoc test for multiple comparisons, to evaluate intergroup differences. Statistical significance was defined as *p* < 0.05. In all figures, the number of replicates (n) for each experiment. Data are presented as box plots depicting the median, first and third quartiles, and minimum and maximum values.

## 3. Results

### 3.1. Radio-Tolerance of Worms Fed with ΔybfQ Bacteria

A dose–response relationship between UV-B radiation and worm growth inhibition was initially established. As shown in Figure 1B, worms exhibited uniform growth inhibition at the dose of 800 J/m^2^. The worms were fed various *E. coli* mutant strains from the Keio collection. Following exposure to 800 J/m^2^ of UV-B radiation, worm radio-tolerance was evaluated based on the degree of growth inhibition. A total of 65 positive strains were preliminarily screened, and among these, a strain carrying a deletion of the *ybfQ* gene was selected for further investigation. The *ybfQ* gene is part of the *ybf* gene cluster, which also contains the *ybfA*, *rhsC*, *ybfB*, *ybfO*, *ybfC*, and *ybfL* genes. In addition to the *ybfQ* gene, the mutation in the *ybfA* gene also demonstrated significant radio-protective effects (Figure 1C). In the aforementioned and subsequent experiments, BW25113, a parental strain of the Keio collection, was utilized as a control. No significant differences in body growth, reproduction, motility, and foraging ability were observed in worms fed either BW25113 or the standard OP50 strain (Figure S1B–E). The radio-tolerance in worms conferred by *ΔybfQ* feeding was also observed across UV-B doses ranged from 600–1000 J/m^2^ (Figure 1D). Furthermore, we investigated the effect of worm ages on *ΔybfQ-*mediated radio-tolerance and found that *ΔybfQ* conferred more pronounced protection to worms irradiated at ages of 16 to 24 h (Figure 1E). Therefore, in subsequent radiation experiments, we predominantly adopted 24-h-old worms (L3 larval). Additionally, we assessed the impact of *ΔybfQ* feeding on worm productivity by measuring their brood size. Following UV-B radiation, *ΔybfQ*-fed worms exhibited significantly lower progeny production inhibition than those fed BW25113 (Figure 1F). To further verify whether the *ybfQ* gene mutation is essential for host worm radio-tolerance, we reintroduced wild-type *ybfQ* gene expression in both *ΔybfQ* and BW25113 via plasmid transfection. The restoration of wild-type *ybfQ* gene expression in *ΔybfQ* substantially reduced worm radio-tolerance in terms of body growth and reproduction; however, no significant alterations were observed when expressing the wild-type *ybfQ* gene in BW25113 (Figure 1G,H). These results suggest that the mutation in the *ybfQ* gene is primary determinant of host radio-tolerance.

Given that UV-B is a form of non-ionizing radiation, we also explored the potential protective effect of Δ*ybfQ* against ionizing radiation. To this end, worms were exposed to 50 Gy of X-ray radiation and 100 Gy of carbon ion radiation. We found that feeding Δ*ybfQ* significantly enhanced worm tolerance to both types of radiation (Figure S1F–I).

### 3.2. Pathways Involved in ΔybfQ-Mediated Worm Radio-Tolerance

**Figure 2:**
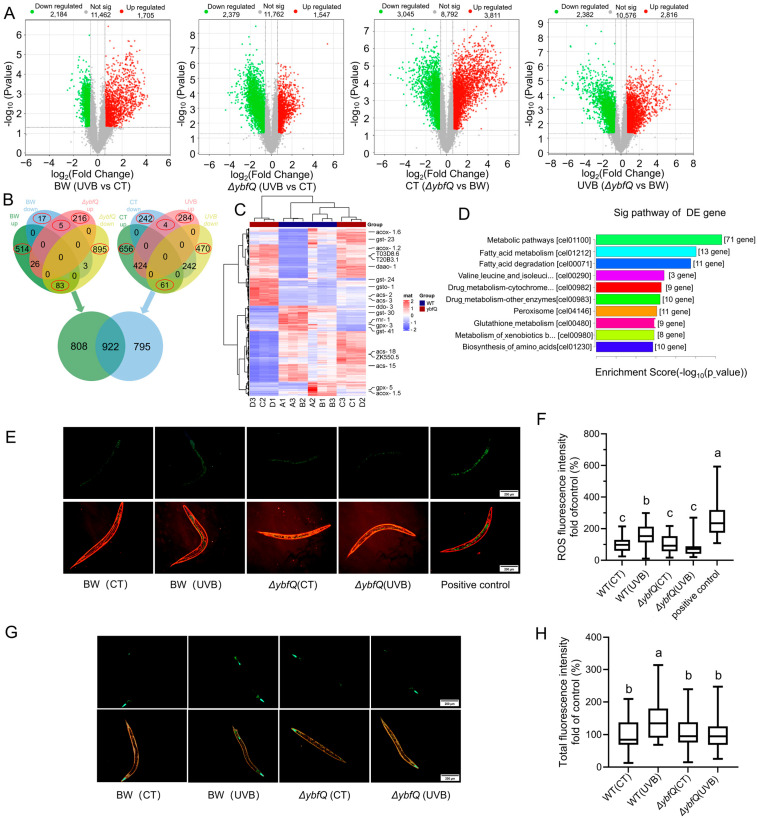
Transcriptome and oxidative stress in worms fed *ΔybfQ* bacteria. (**A**) Volcano plot illustrating differentially expressed genes between the indicated groups; (**B**) Venn diagram of differentially expressed genes. (**C**) Heat map of 2525 differentially expressed genes identified by Venn diagram analysis; (**D**) KEGG enrichment analysis of differentially expressed genes; (**E**,**F**) Quantification of ROS levels in worms using fluorescent probe DCFH-DA (n = 5); (**G**,**H**) Assessment of oxidative status in worms via the expression of the *sod-3::gfp* reporter gene (strain CF1533) (n = 5). BW stands for the BW25113 strain. Significant differences (*p* < 0.05) among experimental groups are indicated with different letters.

To elucidate the mechanisms underlying Δ*ybfQ*-mediated radio-tolerance in worms, we conducted a comprehensive transcriptomic analysis comparing *ΔybfQ*-fed worms with those fed BW25113. Under non-radiation conditions, feeding *ΔybfQ* resulted in the upregulation of 1547 genes and downregulation of 2379 genes compared to feeding BW25113. Upon UV-B radiation, *ΔybfQ*-fed worms exhibited upregulated expression of 2816 genes and downregulated expression of 2382 genes compared to those fed BW25113 (Figure 2A). For the worms fed BW25113, UV-B radiation led to the upregulation of 1705 genes and downregulation of 2184 genes compared to controls. KEGG enrichment analysis revealed that upregulated genes were predominantly enriched in peroxisome and metabolism pathways, while downregulated genes were associated with damage repair pathways (Figure S2A,B). In *ΔybfQ*-fed worms exposed to UV-B radiation, there was an upregulation of 3811 genes and downregulation of 3045 genes compared to control worms. To further identify genes involved in Δ*ybfQ*-mediated worm radio-tolerance, we utilized a Venn diagram approach to analyze differentially expressed genes across four conditions: *ΔybfQ:non-UV-B*, *ΔybfQ:UV-B*, *BW25113:UV-B*, and *BW25113:non-UV-B.* This analysis identified a total of 2525 differentially expressed genes (Figure 2B,C). KEGG enrichment analysis indicated that these genes were primarily enriched in metabolic processes, including fatty acid metabolism, drug metabolism, and peroxidase and glutathione metabolism (Figure 2D). Notably, peroxidase and glutathione metabolisms are closely linked with oxidative stress responses, and their expression patterns are specially illustrated in Figure S2C,D.

### 3.3. Impact of ΔybfQ Feeding on Oxidative Stress in Worms

The involvement of peroxidase and glutathione metabolisms suggests a potential link between oxidative stress mitigation and *ΔybfQ* feeding (Figure 2D). To test this hypothesis, we measured reactive oxygen species (ROS) levels in worms fed *ΔybfQ* and BW25113 using the fluorescent probe DCFH-DA. The results revealed that BW25113-fed worms exhibited a marked increase in ROS levels following UV-B radiation compared to non-irradiated controls. Enhanced fluorescence was observed throughout the entire body, particularly in the head and gut regions. Conversely, *ΔybfQ*-fed worms showed no change in ROS levels post-UV-B radiation (Figure 2E,F). Additionally, we evaluated the expression of superoxide dismutase-3 (SOD-3) using a worm strain transgenic for the *sod-3::gfp* reporter gene. SOD-3 mainly catalyzes the conversion of superoxide into oxygen and hydrogen peroxide [46,47]. In this strain, GFP expression is predominantly localized to the head and tail regions. Following UV-B radiation, worms fed BW25113 demonstrated significantly increased fluorescence intensity in the head and tail regions, with occasional expression detected in the vulva. In contrast, worms fed *ΔybfQ* maintained fluorescence intensity and distribution similar to those of non-irradiated controls (Figure 2G,H). These findings indicate that *ΔybfQ* feeding effectively mitigates UV-B-induced ROS in worms.

### 3.4. Involvement of ΔybfQ Metabolites in Modulating Worm Radio-Tolerance

To determine the specific changes in Δ*ybfQ* bacteria that potentially contribute to the enhanced radio-tolerance in worms, we conducted a comprehensive analysis of multiple phenotypic characteristics of *ΔybfQ*. First, no significant differences were observed in growth rates and cell size between *ΔybfQ* and BW25113 (Figure S3A–C). Second, worms exhibited identical chemotaxis behaviors toward both *ΔybfQ* and BW25113 (Figure S3D). Third, dietary intake patterns were similar when fed *ΔybfQ* or BW25113, as evidenced by the comparable pharyngeal pumping rates (Figure S3E). Collectively, these results suggest that the enhanced radio-tolerance in worms might not be attributable to alternations in bacterial phenotypic characteristics.

**Figure 3:**
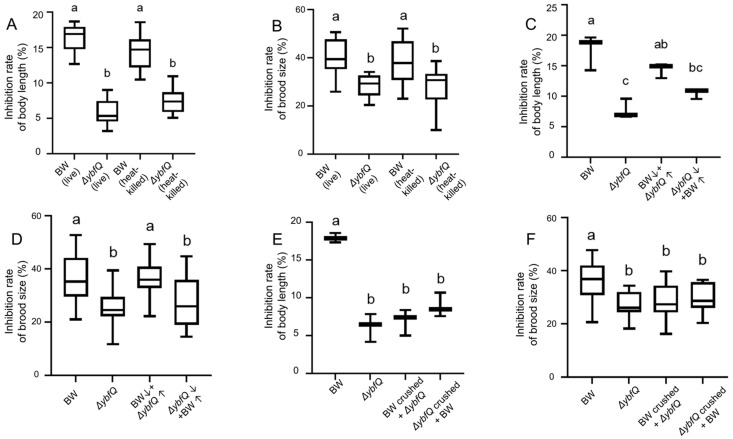
The role of Δ*ybfQ*-derived metabolites in modulating radio-resistance in host worms. Effects of feeding heat-killed bacteria on radio-resistance, as measured by worm growth inhibition (**A**) (n = 6) and brood size (**B**) (n = 6); Effects of bacterial supernatant exchange on radio-resistance, as assessed by worm growth inhibition (**C**) (n = 3) and brood size (**D**) (n = 5); Effects of bacterial homogenate administration on radio-resistance, evaluated through worm growth inhibition (**E**) (n = 3) and brood size (**F**) (n = 5). BW stands for the BW25113 strain. Significant differences (*p* < 0.05) among experimental groups are indicated with different letters.

We further reasoned that the enhanced radio-tolerance observed in worms could be from altered metabolism in *ΔybfQ.* To test this hypothesis, we fed worms with heat-killed bacteria, and likewise observed the enhanced worm radio-tolerance, with gentle reduction compared to living bacteria in terms of growth inhibition. This suggests that metabolites produced by *ΔybfQ* bacteria might play a crucial role in conferring radio-tolerance to host worms (Figure 3A,B). To further validate this observation, we performed supernatant exchange experiments. Feeding worms with a mixture of *ΔybfQ*-conditioned supernatants and BW25113 did not alter their radio-tolerance compared to those fed BW25113 alone. Similarly, feeding worms a mixture of BW25113-condtioned supernatants and *ΔybfQ* did not change their radio-tolerance compared to those fed *ΔybfQ* alone (Figure 3C,D). These findings suggest that the metabolite(s) responsible for worm radio-tolerance might primarily retain within bacterial cells rather than being secreted into the extracellular environment. However, this raises an additional question regarding whether worm radio-tolerance is due to the absence or overproduction of specific metabolite(s) in *ΔybfQ*. To address this, we conducted a bacteria homogenate experiment. Feeding worms with a mixture of *ΔybfQ* homogenate and BW25113 bacteria significantly enhanced their radio-tolerance compared to those fed BW25113 alone, suggesting that enhanced accumulation of specific metabolite(s) in *ΔybfQ* may be essential for the enhanced worm radio-tolerance (Figure 3E,F).

### 3.5. Contribution of Overproduced Isoscoparin in ΔybfQ to Worm Radio-Tolerance

**Figure 4:**
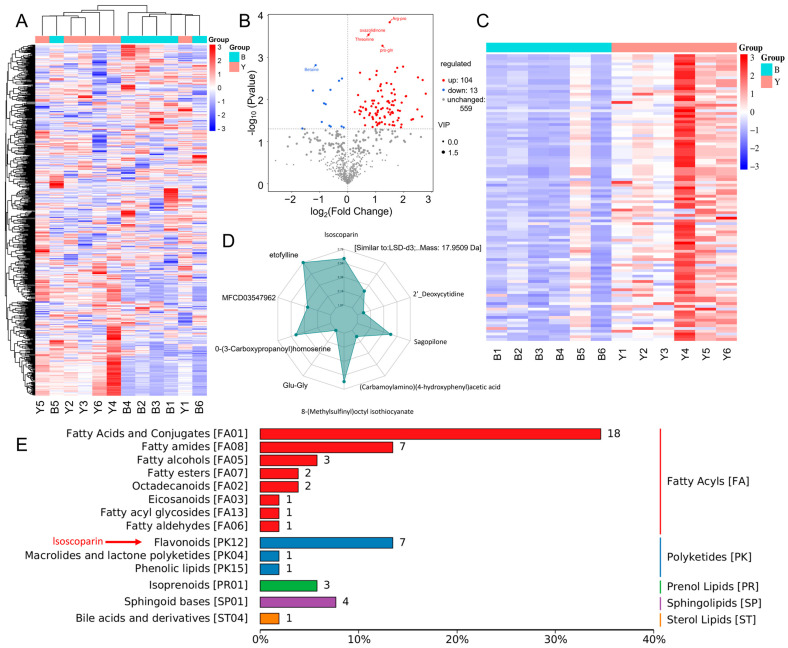
Metabolome in Δ*ybfQ* and BW25113 (BW) strains. (**A**) Heatmap illustrating the distribution of detected metabolites; (**B**) Volcano plot depicting the statistical significance and fold change of 676 metabolites; (**C**) Heatmap showing 104 metabolites with elevated levels in *ΔybfQ*; (**D**) The radar chart highlighting the top 10 metabolites with the largest absolute log2FC values; (**E**) Summary of the classification of these metabolites based on the LIPID MAPS database.

To identify the specific metabolite(s) responsible for worm radio-tolerance, we conducted a non-targeted metabolomics analysis on bacterial cultures. Six replicates of both BW25113 and *ΔybfQ* were analyzed using the BioMarker Company’s platform. A total of 725 peaks were detected, with 676 successfully annotated (Figure 4A). Among these, 117 metabolites exhibited differential abundance between the two groups, including 104 upregulated and 13 downregulated metabolites (Figure 4B). Based on the findings presented in Figure 3E,F, it is reasonable to infer that one or more of the 104 upregulated metabolites may be associated with the enhanced radio-tolerance observed in worms (Figure 4C). Next, we canonically identified the top ten metabolites based on their largest absolute log_2_FC values for visualization in a radar chart (Figure 4D). Among them, isoscoparin stood out as sole compound with antioxidant properties (flavonoid class), with a 2.5-fold increase in abundance (Figure 4D and Table S1). Given the repressive effect of *ΔybfQ* on ROS in worms (Figure 2E–H), we reasoned that isoscoparin might serve as a candidate metabolite for enhancing worm radio-tolerance. This raised the question of whether the administration of isoscoparin alone could replicate the protective effects conferred by *ΔybfQ* feeding.

**Figure 5:**
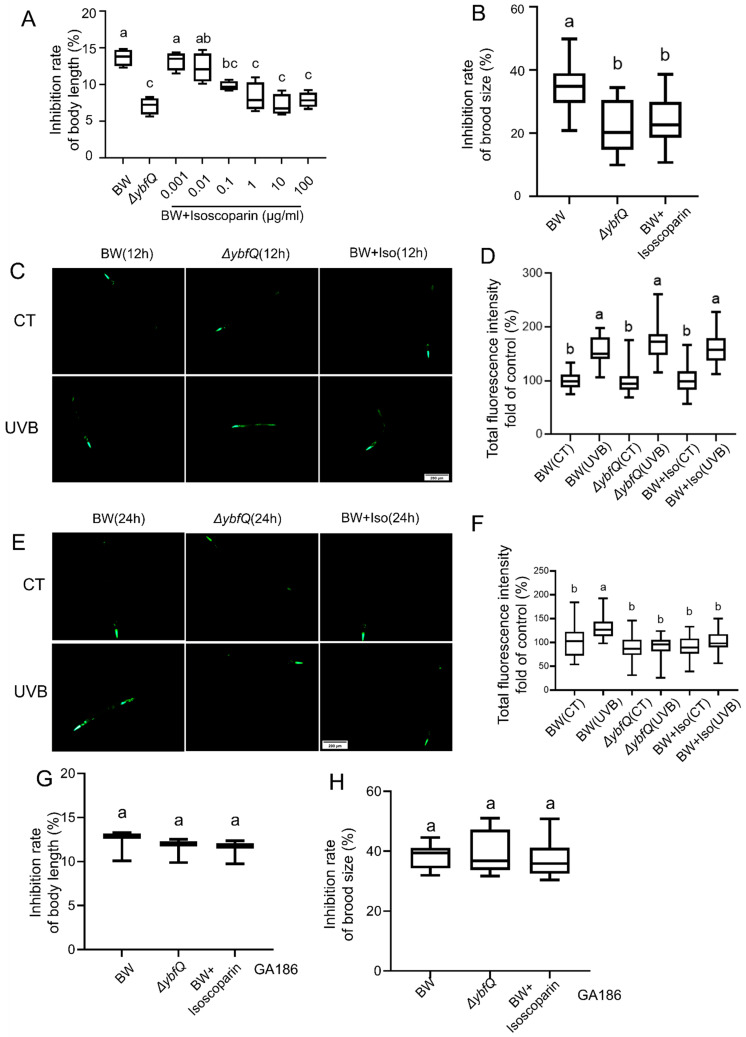
Role of isoscoparin in modulating worm radio-resistance. (**A**) Dose–response relationship between isoscoparin concentration and UV-B-induced inhibition of worm growth (n = 4); (**B**) Effect of isoscoparin (10 μg/mL) on worm brood size (n = 5); (**C**,**D**) Assessment of oxidative stress in worms 12 h after irradiation via expression level of the *sod-3::gfp* reporter gene (strain CF1533) (n = 6). (**E**,**F**) Assessment of oxidative stress in worms 24 h after irradiation via expression level of the *sod-3::gfp* reporter gene (strain CF1533) (n = 6). Effect of feeding *ΔybfQ* and isoscoparin treatment on radio-tolerance in *sod-3* mutant worms, as measured by worm growth inhibition (**G**) (n = 3) and brood size (**H**) (n = 5). BW stands for the BW25113 strain. Significant differences (*p* < 0.05) among experimental groups are indicated with different letters.

For this purpose, we exogenously supplemented the culture medium with isoscoparin derived from Gentiana plants and worms were fed BW25113. The administration of isoscoparin significantly alleviated UV-B-induced worm growth inhibition in a concentration-dependent manner (Figure 5A). At the concentration of 1 μg/mL, isoscoparin treatment exerted a protective effect comparable to that observed in *ΔybfQ*-fed worms. The maximal protective effect was achieved at 10 μg/mL, beyond which no further enhancement was observed (Figure 5A,B). Worms fed ΔybfQ bacteria and treated with isoscoparin exhibited an upregulated expression of *sod-3::gfp* at 12 h post-irradiation compared to those fed BW25113, and a more rapid decrease at 24 h post-irradiation. These results suggest that isoscoparin might function as either a direct antioxidant or a regulator of SOD-3 (Figure 5C–F). The latter hypothesis was supported by the observation that the protective effect of isoscoparin was nullified in SOD-3 deficient worms (GA186) (Figure 5G,H).

**Figure 6:**
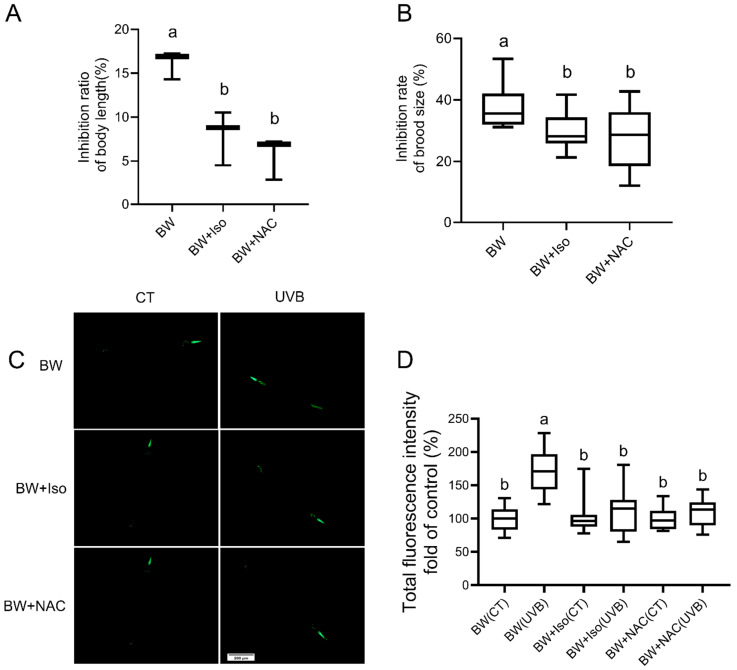
The role of NAC in modulating worm radio-tolerance. (**A**) Effect of NAC on worm body growth inhibition (n = 3); (**B**) Effect of NAC on worm brood size (n = 5); (**C**,**D**) Assessment of oxidative stress in worms via expression level of the *sod-3::gfp* reporter gene (strain CF1533) (n = 6). Significant differences (*p* < 0.05) among experimental groups are indicated with different letters.

Next, we compared the protective effects of isoscoparin and N-acetylcysteine (NAC), a well-established antioxidant compound. Both compounds exhibited comparable radio-protective effects with respect to body growth, reproduction, and antioxidant capacity (Figure 6A–D).

### 3.6. Interaction of Isoscoparin and ΔybfQ with Worms

**Figure 7:**
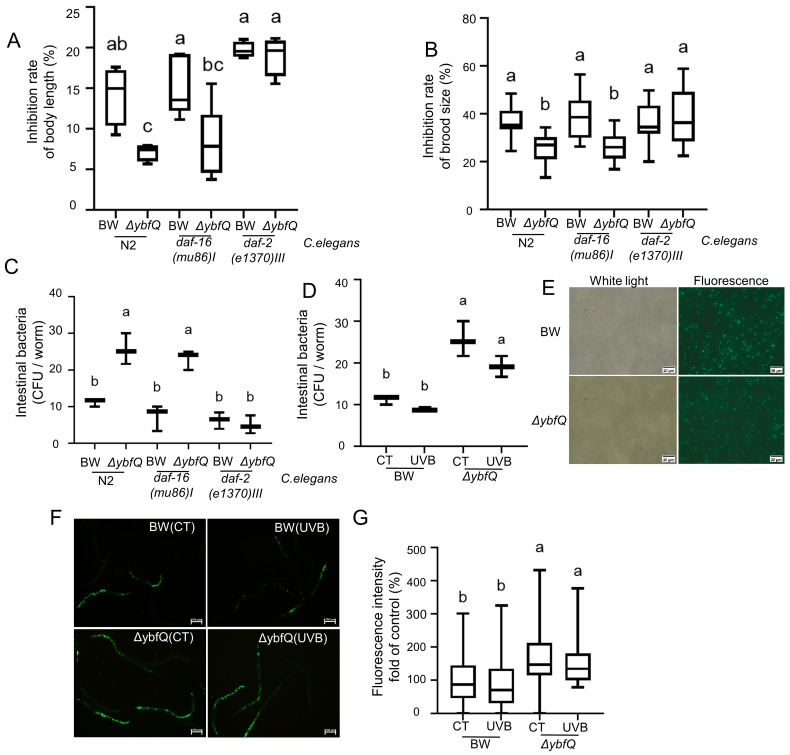
Role of worm immunity in *ΔybfQ*-mediated radio-tolerance. (**A**,**B**) Effect of feeding *ΔybfQ* and isoscoparin treatment on radio-tolerance in *daf-2* and *daf-16* mutant worms, as measured by worm growth inhibition (**A**) (n = 4) and brood size (**B**) (n = 5); (**C**) Quantitative assessment of bacterial colonization in *daf-2* and *daf-16* mutants through clone survival assay (n = 3); (**D**) Quantification of bacterial colonization within worm gut through clone survival assay (n = 3); (**E**) Visualization of bacterial colonization in worms using a GFP marker for BW25113 (BW) and *ΔybfQ* strains; (**F**,**G**) Quantitative analysis of bacterial colonization levels in worm using a GFP marker for BW and *ΔybfQ* (n = 6). Significant differences (*p* < 0.05) among experimental groups are indicated with different letters.

The insulin/insulin-like growth factor-1 signaling (IIS) pathway has been reported to participate in the radiation response [48]. To determine its role in the worm radio-tolerance conferred by isoscoparin treatment and *ΔybfQ* feeding, we evaluated the effects of these two interventions on the radio-tolerance of worms with mutations in the *daf-2* or *daf-16* genes. Specifically, *daf-2* gene mutations constitutively activate the IIS pathway, while *daf-16* gene mutation results in its functional impairment [49]. The results revealed that *daf-16* worms retained enhanced radio-tolerance following isoscoparin treatment and *ΔybfQ* feeding (Figure 7A), suggesting that they might operate independently of the IIS pathway. However, *daf-2* worms exhibited reduced radio-tolerance specifically upon *ΔybfQ* feeding, but not after isoscoparin treatment (Figure 7B).

We compared bacterial colonization levels in the guts of *daf-2* and *daf-16* worms and observed a significantly reduced colonization of *ΔybfQ* in *daf-2* worms, but not in *daf-16* worms (Figure 7C). This result suggests a potential link between worm radio-tolerance and the gut colonization capacity of *ΔybfQ*. Furthermore, we evaluated the gut colonization efficiency of BW25113 and *ΔybfQ* in N2 worms and found that *ΔybfQ* achieved higher viable counts within worm guts than BW25113 (Figure 7D). This observation was further supported by a bacterial GFP-based assay. Using GFP-labeled BW25113 and *ΔybfQ* strains, we assessed their colonization ability in pharyngeal-deficient worms (DA1116) (Figure 7E). As shown in Figure 7F,G, *ΔybfQ* exhibited a greater capacity for gut colonization compared to BW25113. Notably, bacterial GFP expression alone did not affect worm radio-tolerance, and the enhanced colonization of *ΔybfQ* was not due to changes in worm dietary behavior (Figure S4A,D). These findings suggest that, in addition to the accumulation of intracellular isoscoparin, the increased gut colonization of *ΔybfQ* might represent another contributing factor to enhancing worm radio-tolerance.

## 4. Discussion

This study identified the *ΔybfQ* strain and elucidates its radio-protective mechanism in host worms. We determined that the *ybfQ* gene mutation leads to the accumulation of isoscoparin, which acts as a key mediator of this protection. Our findings provide a clear contrast to the complex gut ecosystems of human and other mammals, where research primarily focuses on the impact of overall community structure on the host. Our work establishes a direct causal link between bacterial gene mutation and enhanced host radio-tolerance. Moreover, the protective effect was recapitulated by administering the plant-derived analog of isoscoparin. These results underscore the potential of using engineered probiotics or their bioactive metabolites as novel radioprotective agents for human in high-radiation environments.

However, the protective effects of *ΔybfQ* are influenced by both abiotic and biotic factors. Our results showed that the protective effects of *ΔybfQ* are most pronounced within the doses range of 600–1000 J/m^2^, with no significant protection observed at either lower or higher doses. At low-dose irradiation (≤400 J/m^2^), the intrinsic repair mechanisms in worms appear sufficient to manage DNA damage, as evidenced by minimal growth inhibition and even hermetic effects at 200 J/m^2^ (Figure 1D). Conversely, UV-B doses exceeding 1000 J/m^2^ resulted in more severe damage (Figure 1D), suggesting that the isoscoparin produced by *ΔybfQ* might be insufficient to counteract thus extensive damage. Furthermore, the protective effects of *ΔybfQ* are dependent on the developmental stage of the worms. While the radio-sensitivity of worms decreased with age (Figure 1E), no significant protective effect was observed in worm younger than 16 h, indicating that larval worms require a certain period to assimilate isoscoparin to develop radioprotection. Notably, when worms aged 32 h and 42 h were exposed to UV-B radiation, *ΔybfQ* feeding failed to provide any protective benefit, which might be attributed to the reduced radio-sensitivity at these developmental stages (Figure 1E).

Flavonoids are a diverse class of polyphenolic compounds, with more than 9000 distinct types identified to date [50,51]. These compounds are widely distributed throughout the plant kingdom. Although bacterial systems have been genetically engineered to enhance flavonoid production [52], the natural biosynthesis of flavonoids in bacteria has not been conclusively demonstrated. Isoscoparin, a specific flavonoid compound, serves as an example of the intrinsic biological functions associated with this compound group [53,54]. In this study, we observed approximately a 2.5-fold increase in isoscoparin levels in *ΔybfQ* (Figure 4D), suggesting that bacteria, particularly *E. coli*, possess the capability to synthesize isoscoparin. Therefore, it is plausible that the *ybfQ* mutation enhances the activity of flavonoid biosynthesis pathway, particularly the branch responsible for isoscoparin synthesis. On the other hand, the *ybfQ* and *ybfA* genes are part of the *ybf* gene cluster, and mutations within this cluster have been shown to reduce bacterial transmembrane transport capacity, leading to the intracellular accumulation of substances [55]. Consequently, it is likely that the mutation in the *ybfQ* gene compromises the exocytosis of isoscoparin, thereby contributing to its intracellular accumulation. Supporting this hypothesis, the transcriptomic analysis of *ΔybfQ* bacteria revealed significant alterations in both metabolic pathways and transport systems (Figure S4E–G). Metabolomics profiling identified 117 differentially abundant metabolites between BW35113 and *ΔybfQ* strain (Figure 4B,C), indicating that the *ybfQ* mutation exerts extensive regulatory effects on bacterial metabolism. In addition to isoscoparin, several upregulated metabolites also exhibit antioxidant properties. Notably, administration of plant-derived isoscoparin alone effectively replicates the protective effects conferred by *ΔybfQ* bacteria, underscoring its bioactive potential. However, the protective efficacy of isoscoparin alone does not exceed that observed following *ΔybfQ* feeding (Figure 5A).

The IIS pathway is an evolutionarily conserved signaling cascade across metazoans and is crucially involved in the regulation of development processes, metabolic functions, and behavioral responses [56,57,58]. Previous studies have established that the IIS pathway plays a critical role in modulating cellular and organismal responses to radiation [48,59]. In the present study, we investigated whether the IIS pathway contributes to the radio-protective effects conferred by isoscoparin administration and *ΔybfQ* feeding in worms. Our results indicate that the protective effects of both interventions are independent of the IIS pathway (Figure 7A,B), suggesting a direct interaction with ROS. However, constitutive activation of the IIS pathway in *daf-2* worms diminished the radio-protective effects of *ΔybfQ* feeding, but not that of isoscoparin treatment. Compared to the BW25113 strains, *ΔybfQ* exhibited enhanced gut colonization in N2 worms (Figure 7D). This increased gut colonization was significantly attenuated in *daf-2* worms (Figure 7C). A similar trend was observed for the BW25113 strain (Figure 7C). Notably, no significant difference in gut colonization levels of *ΔybfQ* was observed between *daf-16* and N2 worms (Figure 7C). Importantly, the radio-protective effects of isoscoparin remained unaffected in *daf-2* worms. Taken together, these results suggest that the radio-protective effect of *ΔybfQ* feeding is partially dependent on bacterial gut colonization, which is attenuated by the constitutive activation of IIS pathway. Accumulating evidence indicates that host immunity plays a regulatory role in bacterial colonization within the gastrointestinal tract [60,61]. Given that IIS has been identified as a key regulator of immune homeostasis in worms, its constitutive activation may enhance worm immune responses, thereby limiting bacterial gut colonization.

In addition to environmental radiation, life on earth is facing challenges from climate change, greenhouse effects, and even harsher conditions of deep space exploration. In these contexts, co-adaptation of the gut microbiota in configuration has emerged as a key strategy for host adaption. Our results suggest that this adaptation could be facilitated by harnessing the genetic modulation of specific bacterial species within the gut ecosystem.

## 5. Conclusions

This study establishes a direct causal link between genetic alterations in gut bacteria and host radio-tolerance. Our findings validate the *C. elegans*-*E. coli* symbiotic model, combined with the bacterial knockout Keio collection, as a powerful platform for uncovering the genetic basis of host-microbe interactions. We demonstrate that targeted modulation of even a limited number of gut bacterial species can serve as an effective strategy to systemically enhance an organism’s capacity to maintain homeostasis against environmental stressors. Translated to a medical context, this work underscores the therapeutic potential of engineered probiotics or defined bacterial metabolites as a novel class of radioprotective agents.

## Data Availability

The transcriptomics and metabolomics data that support the findings of this study have been uploaded to Mendeley Data. https://doi.org/10.17632/856cd46z5m.2; Web link: https://data.mendeley.com/datasets/856cd46z5m/2 (accessed on 19 September 2025).

## Supplementary Materials

The following supporting information can be downloaded at: https://www.mdpi.com/article/10.3390/microorganisms13112626/s1, Figure S1: (A) Schematic representation of bacterial selection; (B–E) Effects of feeding BW25113 (BW) and OP50 sttain on the physiological status of worms, as measured by worm growth (B) (n = 4), brood size (C) (n = 5), head thrashes (D) (n = 5), and foraging ability (E) (n =5); Effects of feeding BW and *ΔybfQ* strain on X-ray-induced radio-tolerance, as measured by worm growth inhibition (F) (n = 3) and brood size (G) (n = 5); H and I) Effects of feeding BW and *ΔybfQ* strain on carbon ions-induced radio-resistance, as measured by worm growth inhibition (H) (n = 3) and brood size (I) (n = 5); Significant differences (*p* < 0.05) among experimental groups are indicated with different letters. Figure S2: Transcriptomic changes in worm fed with BW25113 (BW) and *ΔybfQ* strain. (A) KEGG enrichment analysis of up-regulated genes following UV-B exposure; (B) KEGG enrichment analysis of down-regulated genes following UV-B exposure; (C,D) Expression levels of genes involved in peroxidase and glutathione pathways. Figure S3: Effects of the *ybfQ* gene mutation on bacterial traits. (A) Growth curves of bacterial; (B,C) Morphological changes in bacterial size (n = 6); (D) Worm selection preference for different bacteria (n = 5); (E) Effects of feeding with different bacteria on worm pharyngeal pumping activity (n = 5). Significant differences (*p* < 0.05) among experimental groups are indicated with different letters. Figure S4: (A,B) Effects of GFP-labeled bacteria on worm radio-resistance, as measured by worm growth (A) (n = 3) and brood size (B) (n = 5); (C) Effects of different bacterial diets on the pharyngeal pumping rate of UV-B-exposed worms (n = 5); (D) Bacterial selection by UV-B-exposed worms (n = 5). BW stands for the BW25113 strain; (E) Scatter diagram illustrating the differentially expressed genes between BW and *ΔybfQ* strains; (F) Heatmap showing 101 genes differentially expressed in *ΔybfQ*; (G) KEGG classification analysis of these differentially expressed genes. Significant differences (*p* < 0.05) among experimental groups are indicated with different letters. Table S1: The detail information for the top ten metabolites.

## Abbreviations

*Caenorhabditis elegans* (*C. elegans*), *Escherichia coli* (*E. coli*), Green Fluorescent Protein (GFP), Insulin/insulin-like growth factor-1 signaling (IIS), Kyoto Encyclopedia of Genes and Genomes (KEGG), N-acetylcysteine (NAC), Radiation tolerance (Radio-tolerance), Radiation protective (Radio-protective), Reactive oxygen species (ROS), Superoxide dismutase-3 (SOD-3), bacteria with *ybfQ* gene deletion (*ΔybfQ*), Ultraviolet B (UV-B).
