# Supplementary material for: The Effects of Feeding ybfQ-Deficient Gut Bacteria on Radio-Tolerance in Symbiotic Caenorhabditis elegans: The Key Role of Isoscoparin
Source: Microorganisms. 2025 Nov 19;13(11):2626. doi: 10.3390/microorganisms13112626 (PMC12655312; doi:10.3390/microorganisms13112626)
Supplement: Supplementary file 1 [file microorganisms-13-02626-s001.zip › microorganisms-3973848-supplementary.pdf]

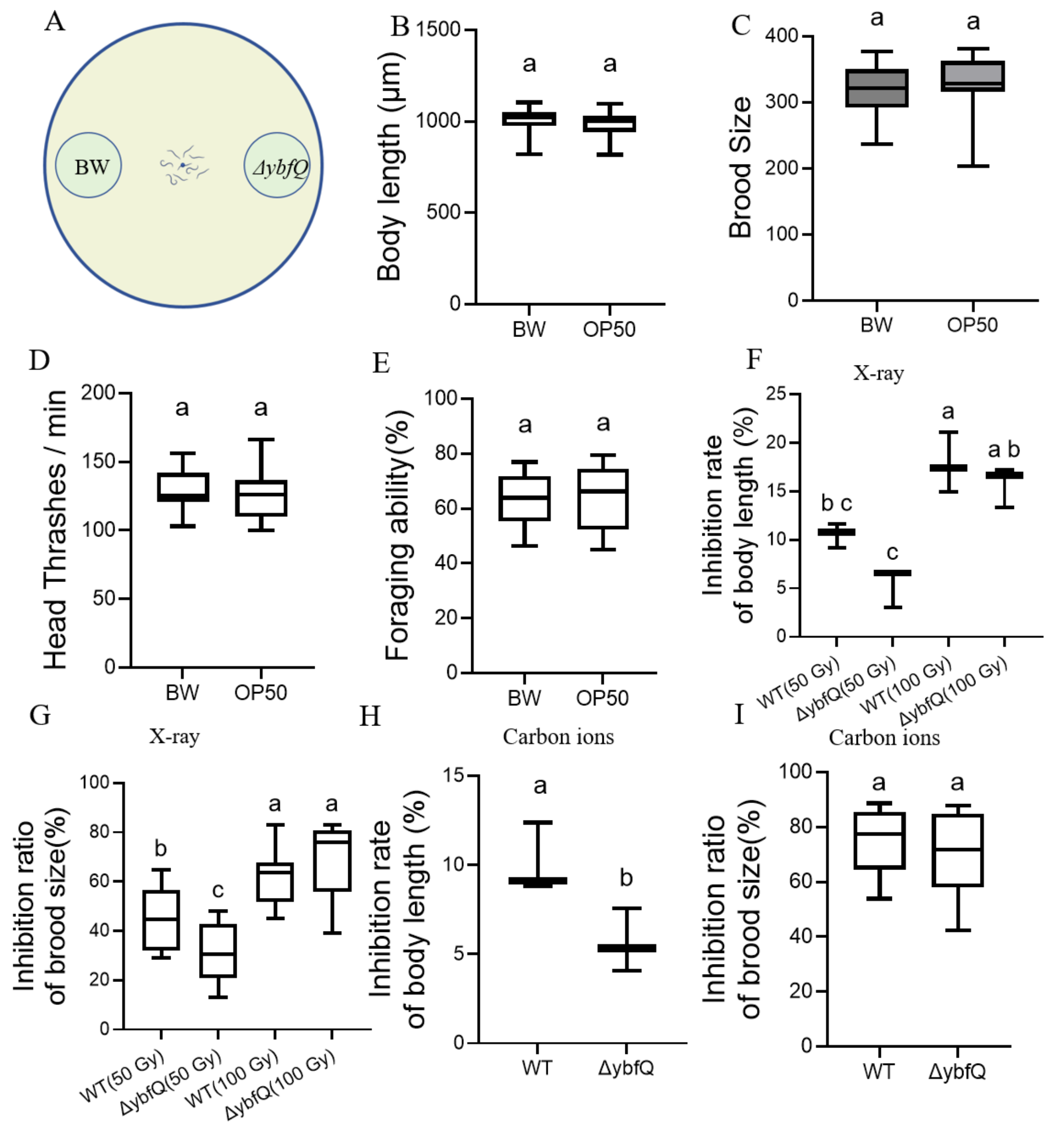

Figure S1. A) Schematic representation of bacterial selection; B - E) Effects of feeding BW25113 (BW) and OP50 sttain on the physiological status of worms, as measured by worm growth (B) (n = 4), brood size (C) (n = 5), head thrashes (D) (n = 5), and foraging ability (E) (n =5); Effects of feeding BW and *ΔybfQ* strain on X-ray-induced radio-tolerance, as measured by worm growth inhibition (F) (n = 3) and brood size (G) (n = 5); H and I) Effects of feeding BW and *ΔybfQ* strain on carbon ions-induced radio-resistance, as measured by worm growth inhibition (H) (n = 3) and brood size (I) (n = 5); Significant differences ($P < 0.05$) among experimental groups are indicated with different letters.

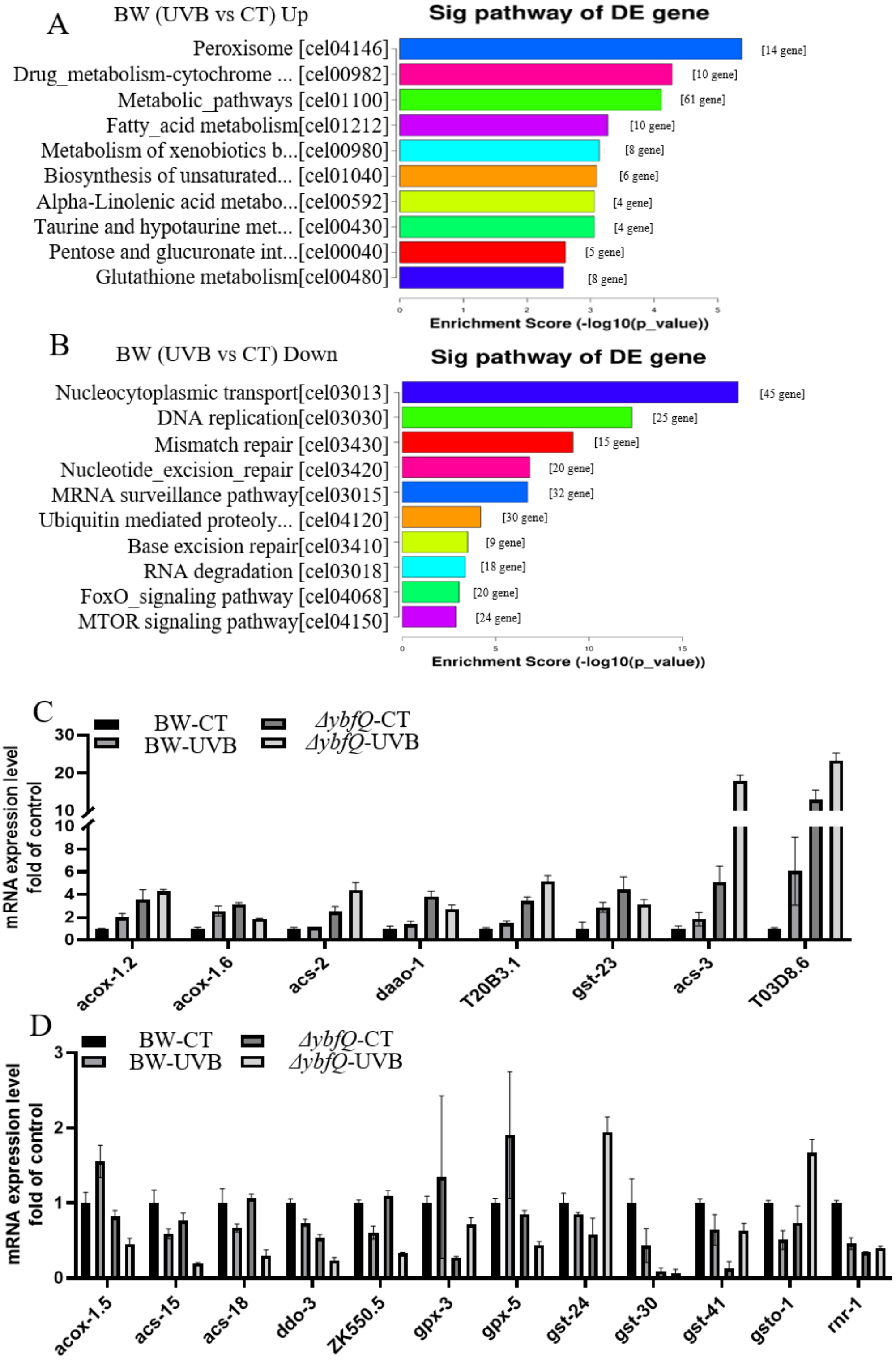

Figure S2. Transcriptomic changes in worm fed with BW25113 (BW) and *ΔybfQ* strain. A) KEGG enrichment analysis of up-regulated genes following UV-B exposure; B) KEGG enrichment analysis of down-regulated genes following UV-B exposure; C and D) Expression levels of genes involved in peroxidase and glutathione pathways.

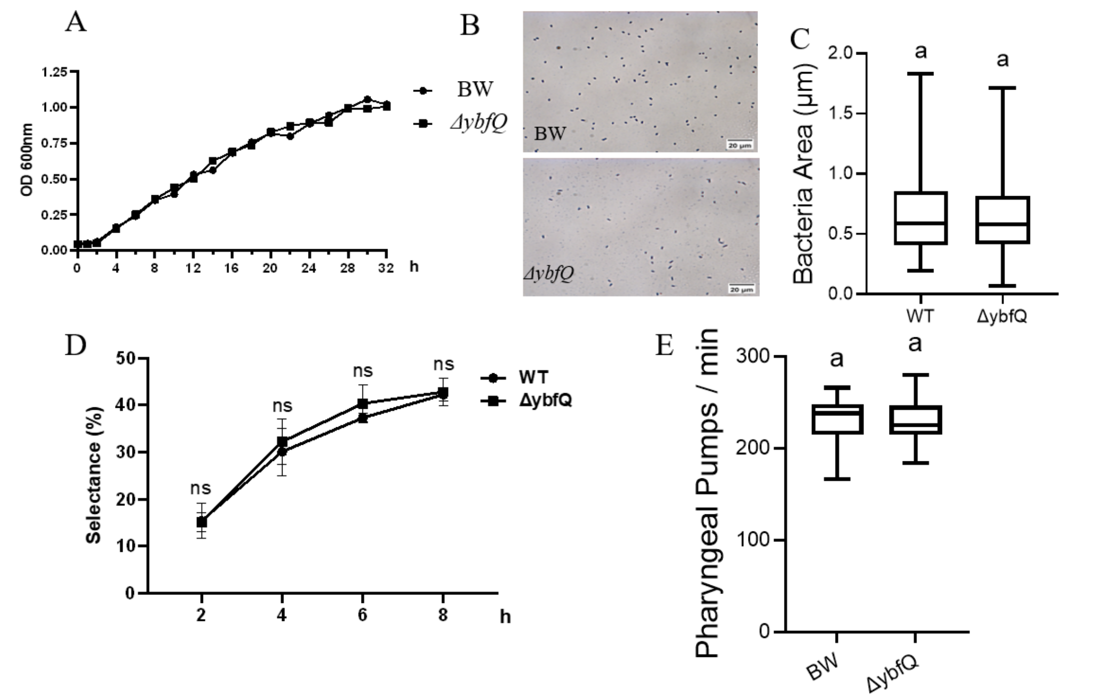

Figure S3. Effects of the *ybfQ* gene mutation on bacterial traits. A) Growth curves of bacterial; B and C) Morphological changes in bacterial size (n = 6); D) Worm selection preference for different bacteria (n = 5); E) Effects of feeding with different bacteria on worm pharyngeal pumping activity (n = 5). Significant differences ($P < 0.05$) among experimental groups are indicated with different letters.

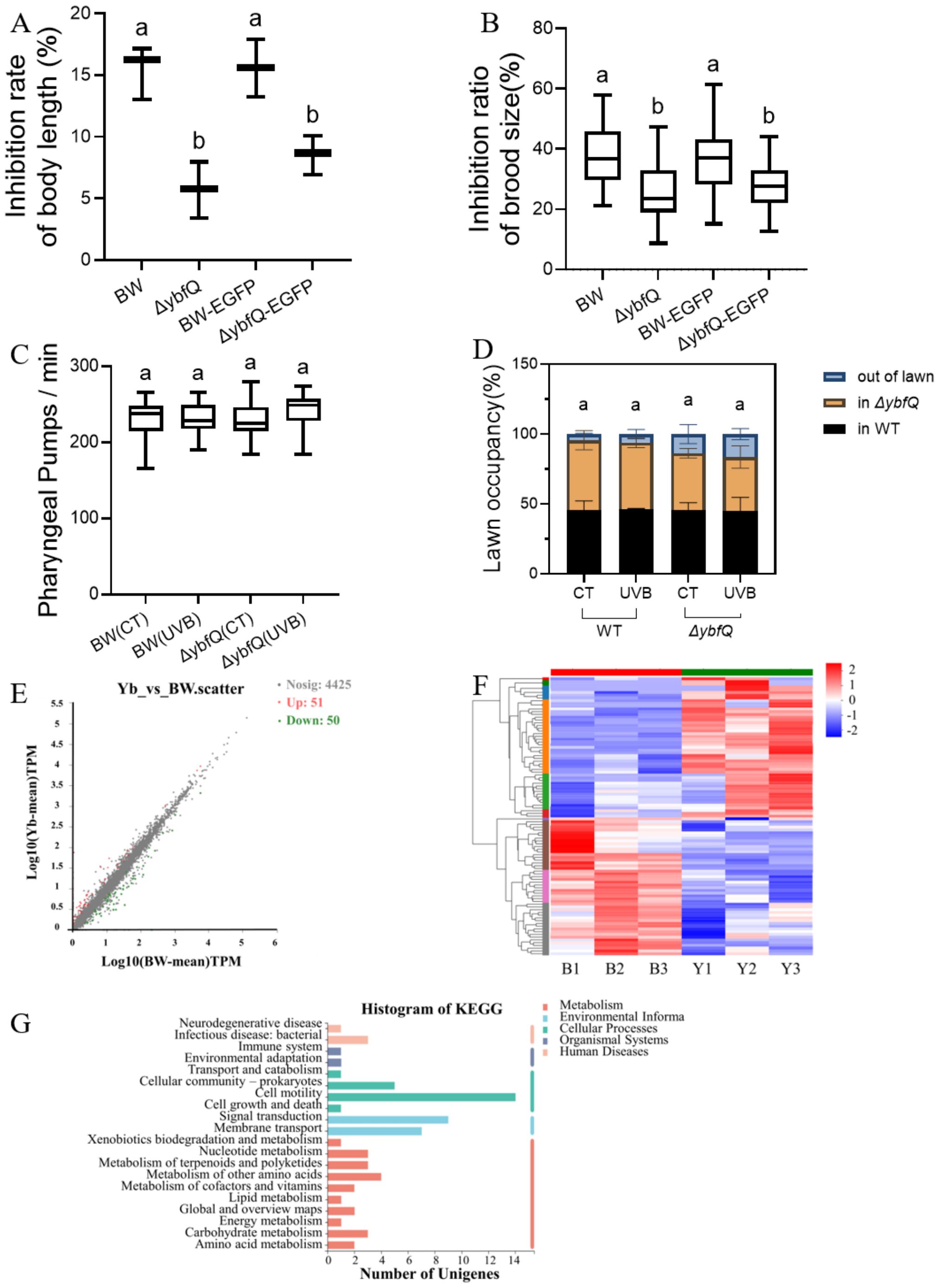

Figure S4. A and B) Effects of GFP-labeled bacteria on worm radio-resistance, as measured by worm growth (A) (n = 3) and brood size (B) (n = 5); C) Effects of different bacterial diets on the pharyngeal pumping rate of UV-B-exposed worms (n = 5); D) Bacterial selection by UV-B-exposed worms (n = 5). BW stands for the BW25113 strain; E) Scatter diagram illustrating the differentially expressed genes between BW and *ΔybfQ* strains; F) Heatmap showing 101 genes differentially expressed in *ΔybfQ*; G) KEGG classification analysis of these differentially expressed genes. Significant differences ($P < 0.05$) among experimental groups are indicated with different letters.

Table S1. The detail information for the top ten metabolites

metabolites	detail information
etofylline	Etofylline (also known as Oxyphylline) is an N-7 substituted derivative of Theophylline. It serves as an anti-hypercholesterolemic agent that effectively reduces total cholesterol levels in the blood. Additionally, it functions as a bronchodilator and may be utilized in research related to asthma management.
8-(Methylsulfinyl)octyl isothiocyanate	An isothiocyanate, has antimicrobial activity and remarkable inhibitory activity against plant growth
lsoscoparin	This compound is a natural flavonoid that exhibits a chemical structure analogous to quercetin. It demonstrates various biological activities, including antioxidant, anti-inflammatory, antibacterial, and anti-tumor properties.
0-(3-Carboxypropanoyl)homoserine	It is a molecule related to bacterial Quorum Sensing (QS) and belongs to Homoserine Lactone (HSL) derivatives
Sagopilone	Sagopilone is a macrolide with potential antineoplastic activity
MFCD03547962	2-Methyl-2,4-pentanediol is a widely utilized diol that is colorless, tasteless, toxic, fully miscible with water, and exhibits high solubility as an organic solvent.
[Similar to:LSD-d3;..Mass: 17.9509 Da]	No found.
(Carbamoylamino)(4-hydroxyphenyl)acetic acid	It's a N-carbamoyl-amino acid.
2'_Deoxycytidine	A deoxyribonucleoside, can inhibit biological effects of Bromodeoxyuridine (Brdu). 2'-Deoxycytidine is essential for the synthesis of nucleic acids, that can be used for the research of cancer.
Glu-Gly	It's a lipid metabolite with a structure resembling that of GABA (γ-aminobutyric acid). It functions as an antagonist of excitatory amino acids.
